# Trajectories of Posttraumatic Growth Among Latvian Parents of Children with Cancer: A Mixed Methods Approach

**DOI:** 10.3390/curroncol32090486

**Published:** 2025-08-30

**Authors:** Inese Lietaviete, Reinis Alksnis, Baiba Martinsone

**Affiliations:** 1 Department of Psychology, University of Latvia, LV-1586 Rīga, Latvia; 2 Department of Science and Technology, University of Latvia, LV-1586 Rīga, Latvia

**Keywords:** childhood cancer, parental stress, post-traumatic growth, post-traumatic stress, latent class analysis

## Abstract

Background: This study explores post-traumatic growth (PTG) among parents of childhood cancer survivors (CCSs), a group often underrepresented in research. Method: A convergent parallel mixed-methods design integrating Bayesian Multilevel Latent Class Analysis and Thematic Analysis was utilized in a longitudinal study involving 58 caregivers (50 mothers, 8 fathers) from the Children’s Clinical University Hospital in Riga. Quantitative data were collected at diagnosis using the Psychosocial Assessment Tool (PAT) and Big Five Inventory-10 (BFI-10). Follow-up assessments post-treatment included the Responses to Stress Questionnaire (RSQ), Impact of Event Scale-Revised (IES-R), and the Post-traumatic Growth Inventory (PTGI). Qualitative data were collected through structured interviews. Results: A 2-class model distinguished parents with low PTG from those with moderate to high PTG. Change in values, detachment from trivial stressors, and acceptance of life emerged as key indicators of growth. PTG was not significantly correlated with overall post-traumatic stress symptoms, but engagement coping strategies showed a positive association with PTG and personality traits like extraversion and openness. Conclusions: The mixed methods approach revealed sample-specific PTG elements not reflected in standardized tools. Initial perceptions of the cancer diagnosis shaped psychological outcomes, with PTG facilitated by adaptive coping, self-reflection, support, emotional disclosure, and psychological struggle. This study offers the first insights into PTG among Latvian parents of CCSs, a previously unexplored area.

## Simple Summary

Parents of children with cancer often face intense stress but may also experience positive psychological changes, known as post-traumatic growth. While such growth has been studied in cancer patients, research on parents remains limited, particularly in Latvia. We followed 58 parents of children treated at the Children’s Clinical University Hospital in Riga, using both questionnaires and interviews. Our study found two groups of parents: those who showed little growth and those who reported moderate to high levels of growth. It was reflected in changed life values, reduced focus on minor stressors, and greater acceptance of life. Growth was not directly related to post-traumatic stress symptoms, but it was positively associated with engagement coping and personality traits such as extraversion and openness. Our findings highlight the importance of adaptive coping, reflection, and social support. These insights can guide future research, inform psychosocial interventions, and support policy aimed at improving parental well-being.

## 1. Introduction

Cancer is one of the leading causes of death among children and adolescents [1]. The chances of surviving a childhood cancer diagnosis vary by country; in high-income nations, over 80% of affected children are successfully treated, whereas in many low- and middle-income countries, the cure rate is below 30%. The five-year survival rate in Latvia for children aged 0 to 14 years diagnosed between 2000 and 2013 was 80.5%, based on approximately 60 newly diagnosed patients per year, according to the EUROCARE-6 population-based study [2]. Although most children diagnosed with cancer are now able to become long-term survivors, parents still face the life-threatening aspects of the illness, which can lead to lasting effects for both them and their child. A significant number of parents report experiencing clinically significant levels of post-traumatic stress symptoms (PTSS) for up to five years following treatment [3,4]. In a Latvian sample of parents of childhood cancer survivors (CCSs), initial stress reactions were closely linked to PTSS following treatment [4]. However, these post-traumatic experiences were also accompanied by various changes, including elements of personal growth.

Post-traumatic growth (PTG) refers to the positive psychological changes that individuals experience during their active struggle after facing extremely challenging crises, traumatic life events, or situations that challenge their core beliefs and assumptions [5,6]. This process can result in personal transformations, shifts in life priorities, an enhanced appreciation for relationships, and a more optimistic perspective on one’s strengths. PTG has been analyzed through various theoretical frameworks, primarily from trauma and personality perspectives [7]. Numerous PTG studies of cancer patients and survivors—particularly breast cancer patients—indicate that PTG is even more prevalent than post-traumatic stress disorder (PTSD) [8,9]. Factors linked to PTG include age, education, economic status, disease threat appraisal, treatment received, social support, and coping strategies. Research shows a curvilinear relationship between PTSD and PTG, where PTG increases alongside PTSD up to a threshold, after which higher PTG correlates with fewer PTSD symptoms [10,11]. This suggests that the relationship between PTSS and PTG may be indirect, mediated by challenges to core beliefs and deliberative or intrusive rumination [12]. However, the interplay remains complex and is not yet fully established [13].

PTG as an outcome can be viewed from cognitive (reorganization of beliefs), emotional (appreciation of life), behavioral (action-focused growth), and biological perspectives [6]. A longitudinal 10-year study of Chinese breast cancer patients identified two PTG profiles: constructive PTG, reflecting genuine transformation through adaptive coping, and illusory PTG, characterized by maladaptive adjustment that creates a positive illusion [14]. PTG can be dynamic and predictive of mental well-being, though it does not mitigate PTSS effects [15]. Some researchers link PTG to personality changes [16], though this remains debated as personality traits are often considered stable in adulthood. Traits such as extraversion, openness, agreeableness, and conscientiousness may predispose individuals to PTG [17]. A review of 70 studies found that coping strategies (e.g., social support, religious coping, positive reframing, reflection) were studied more often than personality traits (e.g., resilience, hardiness, dispositional positive affectivity, gratitude), and the role of the Big Five traits remains unclear [18]. These findings highlight the need for further research into the relationship between coping, personality, and PTG.

Research also shows that parents of CCSs experience notable levels of PTG, often higher than other groups [9,19]. For example, a Swiss study found that parents scored higher than comparison groups in domains such as relating to others, spiritual change, and appreciation of life [20]. Factors such as female gender, older age, higher stress, and greater resilience were positively linked to PTG. Other studies show moderate to high PTG in most parents, with social support and proactive coping serving as key predictors [21,22,23]. PTG has also been linked to enhanced parenting, stronger parent–child relationships, and improved family functioning [24,25]. While sociodemographic and clinical variables appear less relevant, psychosocial factors like social support and coping are strongly associated with PTG. A large review of 154 studies synthesized two conceptual pathways of PTG: a direct pathway through personality traits and coping competencies, and an indirect pathway through the restructuring of core beliefs [26]. Together, these findings underscore the importance of examining PTG in parents of CCSs, particularly using designs that capture longitudinal and latent profiles.

Until recently, PTG in parents of children with cancer has primarily been assessed using overall scale scores, which do not differentiate subgroups or allow tailored recommendations. Person-centered approaches such as latent class analysis (LCA) provide a way of exploring heterogeneity by identifying latent subgroups with distinct PTG patterns [27,28]. Although widely applied in psychosocial research, LCA has rarely been used in pediatric oncology and, when applied, has focused on chemotherapy symptom clusters in children [29,30] or psychosocial outcomes in parents [31,32,33,34]. Only one study has applied latent profile analysis to PTG in CCS parents, identifying three distinct subgroups: ‘Resilience, High Growth,’ ‘Moderate Distress with Growth,’ and ‘Resilience, Low Growth,’ influenced by factors such as gender, socioeconomic status, and treatment intensity [35]. These findings highlight both the heterogeneity of PTG and its implications for family functioning. Our study aims to advance this line of inquiry through a convergent parallel mixed-methods design [36], integrating quantitative profiling with qualitative data on lived experiences. By capturing spontaneous parental accounts of growth [37], this approach provides a richer basis for understanding PTG mechanisms and informing tailored interventions.

This mixed methods study aimed to identify latent classes of PTG among parents of children with cancer in Latvia and qualitatively explore their experiences. We further examined the influence of demographics, personality traits, initial stress reactions, illness cognitions, and coping strategies on class membership, and compared PTSS across classes to inform tailored recommendations for supporting parental well-being during their child’s treatment. The research questions for this study are as follows:

(1) Which latent class model best explains the heterogeneity in PTG outcomes among Latvian parents of CCSs, based on mean differences across identified subgroups?
(2) What are the differences in psychosocial factors (i.e., sociodemographic and personality traits, initial stress reactions, illness cognitions, coping strategies, and PTSS) in the identified latent classes?
(3) What are the parents’ experiences of PTG following their children’s cancer treatment?
(4) How do the quantitative and qualitative sets of results develop a more comprehensive understanding of PTG in parents of CCSs?

## 2. Materials and Methods

### 2.1. Study Design

This article presents data gathered from a longitudinal follow-up study that was part of a broader research initiative involving 120 children diagnosed with cancer and their parents who participated in a psychosocial support program [38] at the Children’s Clinical University Hospital in Riga, Latvia, from 2020 to 2023. With only ~60 pediatric and adolescent cancer cases per year in Latvia (Centre for Disease Prevention and Control), the study population is limited; we therefore sampled all eligible, available cases, capturing nearly the entire national cohort.

**Figure 1:**
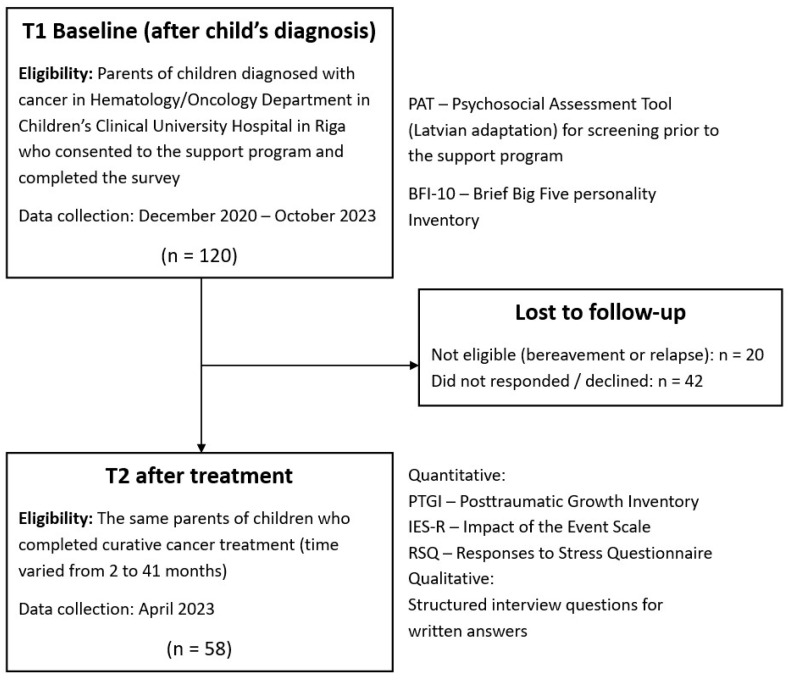
Study design and participant flow.

We conducted a longitudinal, convergent parallel mixed-methods study with two waves of data collection from parents of children with cancer: a baseline quantitative survey after their child’s cancer diagnosis (T1) and a follow-up survey plus qualitative interviews in survivorship (T2), as presented in Figure 1.

Quantitative and qualitative strands were planned a priori and analyzed in parallel, then integrated through triangulation. Reporting follows the GRAMMS guiding mixed methods integration.

### 2.2. Participants and Procedure

Primary caregiver parents of children (0–18 years) diagnosed with cancer (or relapse) and receiving curative treatment (T1) were eligible. Inclusion required agreement to the support program, Latvian proficiency, and decisional capacity for informed consent. We used consecutive, census-style recruitment, inviting all eligible families presenting during the data-collection period; no sampling was performed. At T1, data were collected from the primary caregivers (109 mothers and 11 fathers) of children who had recently been diagnosed with or had relapsed from cancer, with the duration since diagnosis ranging from 1 to 12 months. The ages of the parents varied from 25 to 68 years (mothers: average age = 40.94, *SD* = 6.83; fathers: average age = 39.45, *SD* = 3.64). Among the patients, there were 66 boys and 54 girls, with an average age of 7.13 years (*SD* = 4.73, age range: 0–17 years). The types of childhood cancer diagnosed comprised leukemia and lymphoma (*n* = 74; 62%), brain and spinal cord tumors (*n* = 19; 16%), solid tumors such as sarcoma (*n* = 23; 19%), and other types (*n* = 4; 3%).

At T2 (April 2024), we re-contacted families via email and invited the same parents whose children had completed curative treatment to participate in survey. Parents bereaved between T1 and T2 or facing relapse were not approached, resulting in the exclusion of 20 parents. At T2, data were collected from 58 parents, primarily mothers (*n* = 50; 86%). Their ages ranged from 27 to 54 years (average age = 40.11, *SD* = 3.89). The time since the conclusion of curative treatment varied from 2 to 41 months (average = 14.92, *SD* = 13.45). Initially, the follow-up was intended to last up to 12 months post-treatment, but due to the limited sample size, it was extended to include parents up to 41 months post-treatment who had participated in the baseline study in 2020–2023. Among the patients in this subsample, there were 31 boys and 27 girls, with an average age of 8.95 years (*SD* = 4.12, age range: 3–18 years). The cancer diagnoses in this group comprised leukemia and lymphoma (*n* = 40; 69%), brain and spinal cord tumors (*n* = 5; 9%), solid tumors such as sarcoma (*n* = 12; 20%), and others (*n* = 1; 2%). Families were invited to participate in the second phase of this study via email, with a high response rate of 58%.

Because this was a national cohort within a fixed time window, no formal a priori sample-size calculation was performed; precision and uncertainty are reported with confidence intervals.

### 2.3. Measurements

#### 2.3.1. Screening Measures at T1

- The Latvian version of the Psychosocial Assessment Tool (PAT) [39], derived from PAT 2.0 and PAT 3.1 [40], evaluates psychosocial risks faced by families across five areas, two of which are included in this analysis: ‘Stress Reactions after Diagnosis’ and ‘Family Beliefs’. The ‘Stress Reactions’ subscale addresses the initial stress responses of parents immediately following a diagnosis, including feelings like mood swings, nightmares related to the child’s illness, physical discomfort when discussing the diagnosis, and difficulties relaxing due to heightened stress. The ‘Family Beliefs’ subscale comprises both positive and significantly negative thoughts related to cancer (e.g., “*The doctors will know what to do*” and “*Cancer is a death sentence*”).
- The Big Five Inventory-10 (BFI-10) [41] is a concise self-assessment tool consisting of 10 items (5-point Likert scale) that measures five personality traits: neuroticism, extraversion, openness, agreeableness, and conscientiousness.

#### 2.3.2. Outcome Measures at T2

- The Post-traumatic Growth Inventory (PTGI) [5] is another self-report questionnaire with 21 items (6-point Likert scale) that assesses five domains of PTG: ‘Relation to others’, ‘New possibilities’, ‘Personal strength’, ‘Spiritual change’, and ‘Appreciation of life’.
- The Impact of Event Scale-Revised (IES-R) [42] is a self-report survey for PTSS that includes 22 items (5-point Likert scale) across three subscales: ‘Intrusion’ (which covers intrusive thoughts and nightmares), ‘Avoidance’ (including emotional numbness and avoidance of feelings or situations), and ‘Hyperarousal’ (characterized by irritability and hypervigilance).
- The Responses to Stress Questionnaire (RSQ) [43], the Survivors’ version, is a self-report questionnaire focused on parental coping strategies. It contains 57 items (4-point Likert scale) and is structured into five subscales. At the highest level, coping is categorized as Engagement or Disengagement. Engagement coping is further divided into ‘Primary Control Engagement’ (involving problem-solving, emotional expression, and emotional regulation) and ‘Secondary Control Engagement’ (including cognitive restructuring, positive thinking, and acceptance). ‘Disengagement Coping’ involves strategies like avoidance, denial, wishful thinking, and distraction. Two additional subscales capture involuntary responses to stress: ‘Involuntary Engagement’ (e.g., rumination and impulsive actions) and ‘Involuntary Disengagement’ (e.g., cognitive interference and numbing).
- A short, structured interview with open-ended questions for written answers (e.g., “*It is believed that a child’s cancer diagnosis and prolonged treatment can significantly impact parents. Some of these changes may resonate with you, some you may not like. Have you noticed any differences in yourself compared to before? In what ways have you changed?*”) was employed to explore changes following a child’s cancer treatment. The qualitative questions were informed by the semi-structured Impact of Traumatic Stressors Interview Schedule (ITSIS) [44]. Thematic analysis was conducted to identify and describe the key experiences of growth derived from the qualitative data.

### 2.4. Data Analytics Plan

The methodology employed a triangulated design that incorporated both qualitative and quantitative elements. The integration of results from these two strands facilitated an exploration of the convergence, divergence, and interrelationships between the qualitative and quantitative components. Both qualitative and quantitative approaches were given equal priority; however, the data analysis was conducted independently for each strand. In the interpretation phase, the findings from both strands were synthesized.

#### 2.4.1. Bayesian Latent Class Analysis

Bayesian latent class analysis (LCA) is a statistical modeling technique used to identify unobserved subgroups (latent classes) in a population based on patterns of responses to a given survey’s items. It assumes that conditional on latent class membership, individual item responses are statistically independent, which is known as local independence. This allows the model to capture underlying heterogeneity in response profiles without requiring prior knowledge of group membership.

In this study, Bayesian LCA was used to explore underlying patterns in parental attitudes, beliefs, and perceptions in the context of childhood cancer. The method offers several advantages over traditional LCA: it enables full probabilistic inference, provides posterior distributions for all parameters, and allows the incorporation of prior knowledge through the use of prior distributions (e.g., Dirichlet priors for class proportions and response probabilities) [45]. Model estimation was carried out using Gibbs sampling, as implemented in the BayesLCA package [46] in R. Due to the relatively small sample size, the number of latent classes was restricted to a maximum of two or three in order to ensure model stability and prevent overfitting. While the assumption of local independence may be strong, especially given the potential conceptual correlations between attitudes, beliefs, and coping strategies, it was considered acceptable given the constraints of the data. Model fit was evaluated using the deviance information criterion (DIC) and posterior predictive checks, and models with a low number of classes were found to provide a reasonable balance between simplicity and interpretability. Nevertheless, it should be acknowledged that with a limited number of observations, the granularity of latent class structure may be constrained, and subtle within-class dependencies may remain unmodeled. These limitations were addressed through careful model comparison and sensitivity checks.

#### 2.4.2. Thematic Analysis

We used thematic analysis [47] to capture parents’ experiential accounts of change after treatment and to permit inductive–deductive coding aligned with PTGI domains, facilitating convergent mixed methods integration with our quantitative profiles. Unlike content analysis (frequency-oriented), thematic analysis offers the required flexibility for a national, heterogeneous cohort (sensitive to culture and language) and for constructing binary theme indicators used in integration.

For the qualitative data analysis, the inter-coder (inter-rater) reliability was achieved by having an independent second coder (a clinical psychologist with an academic background). Discrepancies were resolved by discussion with input from an independent clinical psychologist. Agreement rates were calculated for the percentage of codes that were similar, and reliability statistics (Cohen’s κ) met established adequacy thresholds. Rigor was supported by an audit trail, consideration of negative cases, and information adequacy/saturation criteria.

Each theme was converted into a binary variable by assigning a score of 1 or 0 to each individual in the sample, depending on whether or not that individual represented the theme. Qualitative themes were organized alongside PTGI outcomes in joint displays to assess convergence (agreement), expansion, or dissonance. For integration with latent classes, we created binary indicators of key themes (present/absent) to examine associations with class membership. Data were analyzed as frequencies of a theme within a sample by converting them to percentages or proportional means. Insights derived from qualitative data were subsequently used as specific informative priors in constructing latent class indicators.

### 2.5. Ethical Considerations

The research methods and protocols underwent review and received ethical approval from the Institutional Review Board (Nr. 71-43/38). Written informed consent was obtained from parents at baseline.

## 3. Results

This study investigates the long-term psychosocial effects of childhood cancer diagnosis and treatment on parents, emphasizing growth trajectories in the aftermath of cancer treatment. A total of 58 parents, representing 48.3% of the respondents from the initial broader study, completed both the questionnaire and the semi-structured interview.

### 3.1. Descriptive Analyses and Correlation Overview

The results reflect the emotions, needs, and struggles of parents in the transition period after their children’s treatment. Table 1 displays the mean values for each factor derived from the psychosocial measurement instruments, along with the score ranges for the factors on each scale and the internal consistency values calculated using Cronbach’s alpha.

The majority of families readjust to normal life post-treatment, but feelings of isolation and vulnerability persist. Most parents of CCSs involved in this study noted undergoing some form of personal transformation—whether beneficial or adverse—following the completion of their child’s cancer treatment. Notably, 45 parents (78%) acknowledged these changes, while 13 (22%) were unsure or indicated that they did not experience any personal change. Importantly, no significant association was identified between PTG, as measured by PTGI scores, and the overall or cluster-specific PTSS reflected in IES-R scores.

To address the research question about how caregivers’ personality characteristics, illness-related cognitive beliefs, and initial stress responses in T1 are linked to the psychological outcomes (PTSS and PTG) after cancer treatment in T2, a Spearman’s correlation analysis was carried out (Table 2).

In our sample, ‘Neuroticism’ showed positive correlations with PTSS (the IES-R total score) and its ‘Intrusion’ and ‘Hyperarousal’ subscales but a negative link with the PTGI’s ‘Personal Strength’ subscale. Agreeableness was positively related to PTG and ‘Personal Strength’. Initial ‘Stress Reactions’ after diagnosis at T1 correlated significantly with PTSS at T2 after treatment. Cancer-related catastrophic beliefs (e.g., “*Cancer is a death sentence*”) were negatively associated with various aspects of PTG, especially in relationships, new life possibilities, and personal strength, but not with PTSS.

PTG is a multifaceted phenomenon that can appear paradoxical at times. To address this, we explored the diverse trajectories of PTG through LCA and a mixed methods approach that integrates qualitative and quantitative data.

### 3.2. Latent Class Analysis of Parental PTG Following Childhood Cancer

To explore underlying patterns in participant responses, a Bayesian LCA was first conducted to identify unobserved subgroups (latent classes) based on PTGI item responses. This method enabled the probabilistic classification of individuals into distinct latent profiles without assuming pre-defined groupings. Following class identification, posterior probabilities of class membership were extracted for each participant, representing the individual likelihood of belonging to a particular latent class. In the second analytic phase, these posterior probabilities were used as outcome variables in a series of Bayesian logistic regression models to investigate associations between latent class membership and a range of theoretically relevant predictor variables, including sociodemographic characteristics, personality traits, beliefs, and coping strategies. This two-step approach enabled the assessment of how individual differences relate to latent subgroup patterns while accounting for the inherent uncertainty in class assignment. The final stage involved synthesizing the results across models to construct interpretable psychological and behavioral profiles for each latent class.

**Figure 2:**
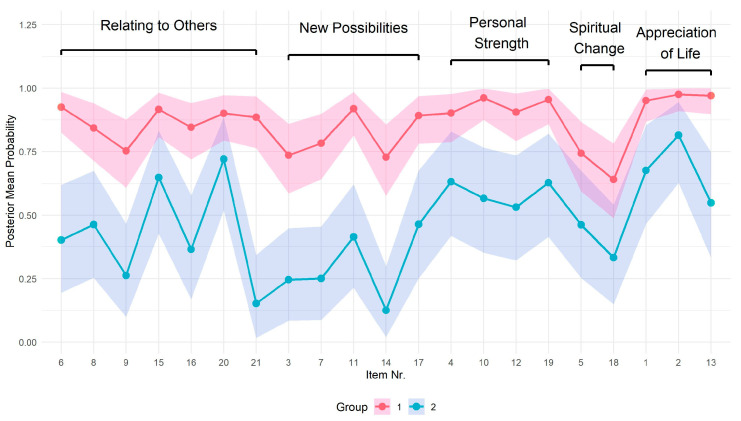
Comparison of latent classes: posterior probabilities with 95% CI. (Group 1 = higher PTG response class; Group 2 = lower PTG response class).

Using Bayesian LCA, a two-class model was estimated to identify underlying respondent subgroups. Figure 2 presents the posterior probabilities of giving a positive response for individuals in each latent class across all PTGI items, along with their corresponding 95% credible intervals. The estimated class proportions indicate that approximately 65% of respondents belong to the higher response group (95% credible interval: 52–75%), while 35% fall into the lower response group (95% credible interval: 22–48%). The higher response group is almost twice as large as the lower response group, which is reflected by the narrower credible intervals for the higher response group.

The main differences in responses were noted in items from the PTGI subscale ‘New Possibilities’. Parents in the lower PTG group tended to disagree with statements such as “*I see new opportunities which wouldn’t exist otherwise*” (item 14) and “*I established a new path for my life*” (item 7). Another distinction between the higher and lower PTG groups emerged in responses related to improved relationships, such as agreement with the statement “*I better accept the need for others*” (item 21).

In the second analytic phase, Bayesian logistic regressions used posterior probabilities to examine links between latent class membership and predictors like demographics, personality traits, coping strategies, cancer-related cognitive beliefs, and parents’ initial stress responses after their child’s diagnosis and PTSS after treatment.

#### 3.2.1. Sociodemographic Factors

The small and heterogeneous sample limited our ability to confirm whether sociodemographic factors predicted latent class membership. Nevertheless, the findings suggest that parents experiencing less frequent shifts in their professional careers after diagnosis were more likely to be categorized in the high PTG class (W = 450.5, *p* = 0.013).

A Bayesian logistic regression was conducted to explore the relationship between the number of siblings and the posterior probability of belonging to the lower response latent class. While the results suggested a possible increase in lower class probability for children with more siblings, all 95% credible intervals included zero, indicating that the associations were uncertain. A Bayesian logistic regression was conducted to assess whether a child’s gender predicted the posterior probability of class membership. The results indicated that, on average, if a child was a girl, the probability of parents being in the lower response group class was slightly higher. However, the 95% credible interval for gender coefficients (−0.52, 0.97) included zero, suggesting that this difference is uncertain.

Additionally, a Bayesian logistic regression was performed to assess whether parental age and marital status predicted membership in the lower-response latent class; however, the results did not reveal associations between these sociodemographic variables and class probability.

#### 3.2.2. Personality Traits

In terms of personality traits, we identified a significant negative correlation between ‘Neuroticism’ and ‘Agreeableness’ within the lower PTG group. This correlation, quantified at −0.47 and reaching statistical significance (df(16) = −2.14, *p* = 0.048), suggests that individuals in this group may experience ambivalence in their relationships with others, particularly under stress. This finding highlights the complex interplay between personality and relational dynamics in the context of trauma recovery.

Although the results did not show direct associations between Big Five personality traits and the probability of class membership, the relationship between personality and PTG is likely mediated by coping mechanisms, such as social support-seeking strategies, positive reappraisal, etc.

#### 3.2.3. Coping Strategies

Our findings suggest that certain coping strategies, as measured by the Responses to Stress Questionnaire (RSQ), are associated with higher levels of PTG. Table 3 presents a summary of the logistic regression models, where the outcome was dichotomized class membership. Among all tested predictors, ‘Primary Engagement’ strategies showed a statistically meaningful association with class probability, as its 95% credible interval did not include zero. All other predictors had wide intervals overlapping zero, suggesting weak or no evidence of association.

**Figure 3:**
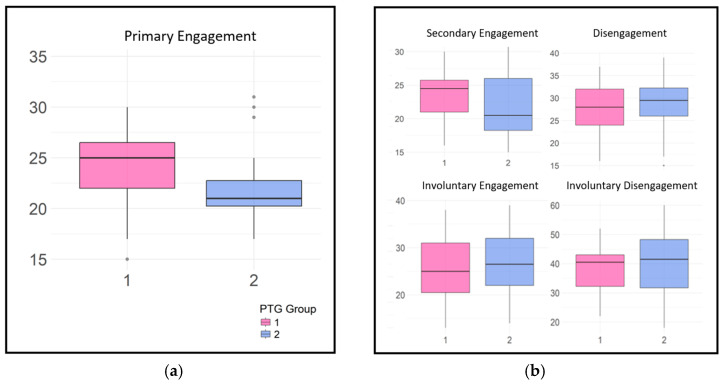
Latent class variations in coping responses: (**a**) primary engagement; (**b**) secondary engagement, disengagement, involuntary engagement, and disengagement.

The higher PTG group (M = 24.54) showed significantly higher ‘Primary Engagement’ coping values than the lower PTG group (M = 22.22) at a 5% significance level (t(34.23) = −1.96, *p* = 0.03). Other coping strategies, such as ‘Secondary Engagement’, ‘Disengagement’, and ‘Involuntary’ coping reactions, did not show statistically significant differences between groups (Figure 3).

This suggests that the way individuals approach and manage stress may play a crucial role in facilitating their growth after traumatic experiences. Overall, these insights contribute to a deeper understanding of the factors influencing PTG and underscore the need for tailored interventions that consider both psychosocial and personality dimensions.

#### 3.2.4. Psychosocial Factors After Diagnosis

None of the tested predictors, measured by the Psychosocial Assessment Tool (PAT) after diagnosis, including perceived support, the child’s emotional state, emotional expressions of family members (e.g., symptoms of depression, anxiety), initial stress reactions of parents after diagnosis, or family beliefs about illness showed a statistically credible association with class membership after treatment. In all cases, the 95% credible intervals for the regression coefficients included zero, suggesting substantial uncertainty about the direction and magnitude of effects. The strongest (yet still non-significant) signal was observed for emotional expressions within the family, but the wide interval (−0.41 to 0.78) indicates high variability in the data (Table 4).

We faced challenges in identifying reliable predictors for determining which parents would belong to the higher PTG class based on their initial psychosocial risk assessment (PAT total score). Notably, two of the five mothers initially classified as having very high psychosocial risk (the clinical tier) ultimately belonged to the higher PTG group after their daughters’ successful treatment, suggesting that early assessments may not fully reflect the complex trajectory of psychological growth.

Analysis of the qualitative data revealed some differences in how parents from distinct PTG classes recalled and articulated their memories of the most traumatic moments experienced during their child’s cancer treatment journey. The most frequent and commonly shared theme across both groups was ‘Diagnosis Shock’ (22% in higher PTG group, 29% of respondents in lower PTG group). Here are two examples from both groups:

Higher PTG group:

*May 19, when the doctor called me in to inform me about the diagnosis. Shock, the inability to accept that this is happening to my child, and that what is about to begin will also become a part of my life. You’re facing something with no known endpoint or measurable distance. A day when you’re left with the thought that you lack the ability to take responsibility for your own tomorrow, or perhaps for the rest of your life.*

(Mother, 46B)

Lower PTG group:

*The very beginning at BKUS, in ward 10, right after the diagnosis was announced and we were transferred to this ward—I didn’t know anyone, I didn’t know the ward rules (mask-wearing, not being able to meet freely, accidentally breaking some rule and getting corrected for it), I didn’t know what to expect—all combined with my own emotions, when I could suddenly start crying out of nowhere (and I really dislike crying when others can see it).*

(Mother, 33B)

Key differences between the groups emerged in the way parents formulated their narratives: overall, the lower PTG group used fewer words, provided less detailed accounts of their emotional experiences, and did not explicitly report ‘Emotional Distress’, which may indicate emotional suppression or avoidance. As one mother from the lower PTG group noted, “*The survey is truly an emotional challenge. To be honest, I really didn’t want to remember and delve into it again, because I’ve made a very deliberate effort to move away from it*” (30A). In contrast, the higher PTG group more frequently provided vivid and detailed recollections of traumatic medical experiences. Additionally, this group showed a higher occurrence of ‘Emotional Distress’ in their narratives of the most traumatic moments, suggesting deeper emotional processing—a factor closely linked to the development of PTG.

A quantitative comparison revealed no statistically significant difference in mean support scores between groups. However, a closer analysis of qualitative data showed that respondents in the higher PTG group reported significantly more diverse sources of support (M = 5.03, SD = 2.19) than those in the lower PTG group (M = 3.81, SD = 1.99), t(32.45) = –2.17, *p* = 0.035. These sources included not only close family (e.g., partner, parents) but also extended networks and professionals (e.g., psychologists, peer groups). In contrast, respondents in the lower PTG group more often mentioned limited or inward-focused support, typically only from immediate family members. This suggests that engagement with a broader support system may facilitate or reflect higher PTG.

#### 3.2.5. Post-Traumatic Stress Symptoms

**Figure 4:**
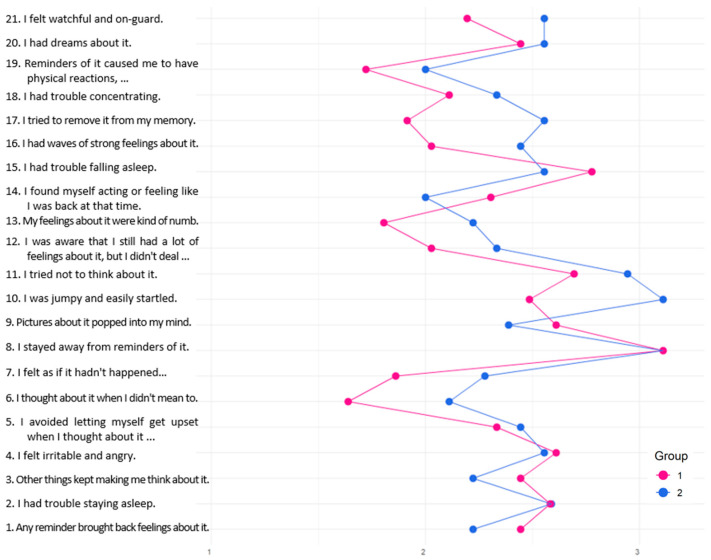
Latent class variations in PTSS based on IES-R items.

No significant differences in overall PTSS were found between respondents in the lower and higher PTG groups. However, as shown in Figure 4, some differences emerged in responses to items from the IES-R subscale ‘Avoidance’. Parents in the lower PTG group were more likely to endorse statements such as “*I felt as if it hadn’t happened or wasn’t real*” (item 7) and “*I tried to remove it from my memory*” (item 17), suggesting a greater reliance on avoidant coping mechanisms.

Qualitative data from narratives describing how parents articulate their bodily stress experiences suggest that those in the higher PTG group not only endured significant stress but also appeared to process it more vividly and openly, consistent with greater emotional engagement, a known contributor to PTG. When symptom frequencies are adjusted for the number of respondents in each group, parents in the higher PTG group more frequently report experiences such as accelerated heartbeat (70% vs. 52% in lower PTG group), muscle tension (54% vs. 38%), rapid breathing (32% vs. 14%), and tend to describe multiple symptoms concurrently. This pattern may indicate a stronger attunement to bodily sensations. Additionally, several intense or panic-related responses—such as “*I thought I would go crazy*” (mother, 15A), “*I called an ambulance*” (mother, 15B), and “*My eye started twitching. It began occurring after the eighth month of treatment*” (mother, 48B)—were reported exclusively by this group, further highlighting a deeper level of emotional and physiological awareness.

Data revealed that PTG does not eliminate the persistent fear of the illness returning. Furthermore, quantitative results from the RSQ in both the lower and higher PTG classes indicate that even 12 months or more after treatment, parents’ primary concerns remained consistent—they continued to worry most about the possibility of cancer recurrence and the long-term side effects of their child’s treatment, including changes in appearance, emotions, behavior, or abilities. Parents in both the higher and lower PTG classes displayed similar patterns of post-treatment worries.

Furthermore, the semi-structured interview revealed that once active treatment ends, parents face a web of interconnected worries. We distinguished six main themes in the narratives. They struggle with (1) the academic catch-up and social reintegration their child faces after long absences from school while simultaneously living with the ever-present (2) fear of relapse, questioning the adequacy of past therapy, fearing the financial burden of renewed treatment, and dreading how to break such news to their child. These medical uncertainties compound (3) parents’ own mental-health challenges: many feel exhausted, anxious, and even stigmatized when clinicians dismiss their personal health complaints as “just stress.” As one mother admitted, “*I’ve started hiding the fact of my child’s illness when I visit my own doctor; everyone blames my health problems on stress and does nothing*” (15B). Long-standing anxiety and depressive symptoms erode their sense of self-efficacy. (4) Family dynamics add further strain as parents juggle the needs of healthy siblings, a working partner, and decisions about future children. At home, they remain (5) hyper-vigilant, worried about overlooking early warning signs or exposing their immunologically vulnerable child to everyday viruses, particularly when kindergarten or primary school resumes. Threaded through all of these issues is (6) a pervasive sense of uncertainty and loss of control that can leave families feeling adrift even after active treatment ends. As another mother noticed: “*Uncertainty creates absolute chaos*” (54A). Unable to foresee what lies ahead, academically, medically, emotionally, or logistically, many parents find that intolerance of the unknown is their chief source of distress.

### 3.3. Mixed Methods Approach to Parental PTG in Childhood Cancer

The integration of quantitative and qualitative data strands provides trauma-specific and idiographic insights into PTG that standardized measures may overlook. This section presents a thematic synthesis of qualitative narratives from parents of CCSs, analyzed to explore perceived personal changes—positive or negative—following their child’s treatment. The analysis aims to reflect the nuanced, individually constructed nature of these experiences. Theme frequencies (Table 5) are presented for each group (number in parentheses), adjusted as a proportional mean to the subsample size.

The themes identified in parents’ narratives about changes they experienced after their child’s cancer treatment largely aligned with the five dimensions of the post-traumatic growth model proposed by Tedeschi and Calhoun [5]. Among these dimensions, ‘Appreciation of Life’ (in 30 narratives (52% of respondents)) and ‘Personal Strength’ (in 23 narratives (40% of respondents)) were the most represented, followed by ‘Relating to Others’ (in 10 narratives (17% of respondents)). Parents in the higher PTG group expressed a wider range of growth-related themes and were more likely to report multiple, interrelated changes across emotional, behavioral, and relational domains, whereas those in the lower PTG group reported proportionally more psychological distress.

Notably, we identified several subthemes that contrasted with growth and may reflect the ongoing struggles experienced by parents. Within the ‘Relating to Others’ domain, the changes were sometimes associated with social withdrawal, shifts in trust, and relational strain. In the ‘Personal Strength’ domain, perceived growth often coexisted with increased anxiety, heightened health-related vigilance, and persistent psychological burden. These patterns were more pronounced in the lower PTG group. However, it was very common for parents to describe both positive and negative outcomes within the same narrative:

“*I’ve become calmer about the big things in life because I’m no longer as afraid. But the joy of life is gone—it no longer feels easy.*”

(G1, 31B)

“*And once again—both difficult experiences [divorce and child’s cancer] shaped the changes in me, as they happened simultaneously. I’ve become much braver, more assertive, and more demanding—yet at the same time, more hesitant when it comes to trust. I’ve become less open, more inwardly reserved, yet simultaneously more empathetic and emotionally calm. I now deeply appreciate the things I once took for granted, value the genuine people around me, and love and protect those who are truly mine even more. As my psychologist said after a full year of sessions, I’ve become much stronger.*”

(G1, 32B)

A noteworthy subtheme that emerged was ‘Reduced Reactivity to Trivial Matters’, identified in 10 narratives (17% of respondents). While this theme reflects a shift in personal values and closely aligns with the ‘Perspective Shift/Change in Values’ subtheme within the ‘Appreciation of Life’ domain—the most frequently represented subtheme in our sample (29%)—its emphasis on inner resilience and the reprioritization of everyday concerns justifies its classification within the ‘Personal Strength’ domain. This placement highlights the nuanced ways in which psychological adjustment may manifest, emphasizing not only cognitive reframing but also behavioral restraint and emotional regulation.

Additionally, a culturally specific subtheme emerged in our research—an increased appreciation for Latvian medical care because of the treatment experience (identified in two narratives). Another emerging subtheme (identified in two narratives) was the desire to help other parents through shared experience, reflecting a form of meaning-making after the cancer-related trauma. As one father admitted: “*I would like to share our family’s experience with other parents as a source of strength and encouragement*” *(G2, A12-2).*

Overall, the findings of our study suggest that participants in the higher PTG group exhibited a broader range of themes related to self-regulation, life appreciation, and relational growth. Shifts in personal values, reduced reactivity to minor stressors, and greater life acceptance were key indicators of growth. These findings extend the ‘Personal Strength’ dimension of the PTG model by highlighting subtle forms of self-regulatory change, beyond traditional notions of resilience. Moreover, our results reveal a nuanced picture of social change. While many participants reported strengthened familial bonds, others described experiences of social withdrawal, distrust, or strained relationships outside their immediate family. This suggests that social transformations following trauma are ambivalent rather than uniformly positive.

Additionally, participants expressed heightened awareness of health and the body, including increased vigilance regarding their children’s health and their own self-care. These experiences straddle the boundary between growth and anxiety and may indicate an emerging dimension of somatic consciousness—a facet not fully captured in Tedeschi and Calhoun’s original PTG model.

Table 6 shows an integrated overview of parents’ PTG experiences by combining quantitative and qualitative findings. On the left, the boxplots display average scores for the five PTGI subscales. Each boxplot represents the interquartile range (the middle 50% of responses, from the 25th to the 75th percentile). On the right, corresponding qualitative responses provide deeper insight.

This mixed methods analysis revealed both convergences and divergences between quantitative scores and qualitative narratives across the five PTG domains, highlighting domain-specific pathways of adaptation among parents following their child’s cancer treatment.

In the ‘Relating to Others’ domain, greater variability—especially in the lower PTG group—suggests divergent relational adjustments. While increased compassion was commonly reported, limited emotional expressiveness points to a tension between relational sensitivity and emotional restraint. Similarly, participants with high ‘Personal Strength’ scores described cognitive–emotional growth, particularly in emotional regulation and value shifts, though unresolved distress—such as anxiety and hypervigilance—remained prominent among lower scorers.

‘Spiritual Change’ and ‘New Possibilities’ showed the widest score dispersion, especially within the lower PTG group. This variability reflects potential spiritual ambivalence and limited engagement with new opportunities. Narratives and item responses indicate a shift toward pragmatic adaptation rather than expansive reinvention, with a stronger emphasis on understanding spiritual matters than on deepening religious faith.

‘Appreciation of Life’ emerged as the most consistently represented and endorsed domain. High scorers described present-moment awareness, value reorientation, and gratitude, though some accounts may reflect social desirability or illusory growth. The co-occurrence of appreciation and existential reflection illustrates the complex interplay between vulnerability and meaning-making.

Overall, our findings support contemporary extensions of the PTG framework that emphasize the integration of struggle and growth, and which view emotional processing as central to the development of PTG, rather than focusing solely on outcome-based indicators.

## 4. Discussion

This study yielded several important insights. First, a two-class latent model effectively distinguished between parents of CSSs with lower versus moderate-to-high levels of PTG, with approximately 65% of participants falling into the higher PTG group. Second, the mixed methods analysis revealed that individuals in the higher PTG group expressed a broader and more nuanced range of themes related to self-regulation, life appreciation, and relational growth. Shifts in core values, reduced reactivity to everyday stressors, and greater acceptance of life’s uncertainties emerged as salient indicators of growth. Third, engagement coping strategies—such as problem-solving, emotional expression to others, and emotion regulation—were significantly associated with PTG and predictive of class membership. Fourth, PTG is not a uniformly positive outcome but a complex, multidimensional process in which emotional processing, meaning reconstruction, and the integration of both struggle and adaptation play central roles.

### 4.1. Comparison to Previous Research and Novelty

To our knowledge, this study is one of only a few that have applied LCA to examine trajectories of growth in parents of CCSs [35] and represents the first such study conducted in Latvia. The emergence of a larger group (65% of respondents) with moderate-to-high PTG aligns with the existing research, which consistently reports elevated PTG levels among this population [25]. In comparison with previous studies on PTG among parents of CCSs, the mean total score reported by Latvian parents (M = 59.32, SD = 17.82), based on the raw sum of the five PTGI dimensions using a 6-point Likert scale, appears relatively elevated—positioned between the lowest and highest PTG levels documented in the literature (with total sum scores from 46.8 [48] to 72.9 [49]). However, several limitations relating to the sample must be acknowledged. Only 48.3% of the original participants from the first phase of this study consented to participate in the follow-up, raising potential concerns about self-selection bias. Observationally, parents with higher PTG were more likely to provide detailed responses and engage more openly with emotionally charged topics. In contrast, those in the lower PTG group tended to be more reserved or avoidant, often contributing minimal information. It is therefore plausible that the sample overrepresents emotionally expressive individuals with higher PTG levels, limiting the generalizability of the findings to the broader population of CCS parents.

Our findings, derived from quantitative and qualitative data integration, confirm that parents, following their child’s cancer treatment, report evident signs of personal growth, particularly within the PTG domains of ‘Personal Strength’ and ‘Appreciation of Life’. However, ongoing debate persists regarding whether self-reported PTG reflects authentic psychological transformation or is instead shaped by perceived growth, potentially driven by mechanisms like positive reinterpretation or self-enhancing bias [50]. Nonetheless, even if such growth is partially illusory, the perceived enhancements in functioning—reflected in shifts in values prioritizing family bonds, reduced sensitivity to minor stressors, recognition of inner strength, and greater acceptance of life—indicate a broader pattern of adaptive adjustment within our sample.

### 4.2. Latent Classes and Correlates of PTG: Personality, Coping, and Social Network

Quantitative findings revealed good internal consistency across outcome measures (PTGI, IES-R, RSQ). While post-traumatic stress and PTSS remained prevalent, they were not significantly associated with PTG scores, suggesting that growth and distress can coexist. This interpretation was supported by qualitative data and further reinforced by an analysis of parents’ concerns following their child’s treatment, which were notably consistent across both latent classes—for example, fear of cancer recurrence emerged as a predominant concern among parents in both the lower and higher PTG groups.

Neuroticism was found to be positively associated with PTSS but not with overall PTG (except for a negative association with the ‘Personal Strength’ subscale), linking higher emotional instability to greater psychological distress following trauma, as has been reported in previous studies [18]. Agreeableness was positively associated with overall PTG, indicating that interpersonal sensitivity, empathy, and cooperation may facilitate growth, potentially through enhanced relational coping or willingness to seek support. Additionally, a significant negative correlation between neuroticism and agreeableness was observed in the lower PTG group, potentially indicating relational strain that may hinder psychological growth in this group. However, personality alone does not fully explain the resources individuals use in response to adversity—the relationship between personality traits and PTG appears to be mediated by coping strategies [17]. Consistent with this notion, our findings suggest that traits such as extraversion, agreeableness, and openness to experience may be associated with PTG through their influence on active coping mechanisms, including self-disclosure, seeking social support, and trying new ways of seeing things.

LCA revealed distinct differences between the lower and higher PTG groups, particularly on the ‘New Possibilities’ and ‘Relating to Others’ subscales. Respondents in the lower PTG group reported limited recognition of new opportunities and were less likely to acknowledge the importance of interpersonal relationships compared to those in the higher PTG group. Bayesian logistic regressions further identified ‘Primary Engagement’ coping strategies (e.g., problem solving, emotional expression, and regulation) as significant predictors of high PTG class membership. This finding is well supported by recent research [25]. Analyzing these results together with the qualitative data strand, we also find differences in the social networks as described in the narratives. While no significant differences emerged in overall support-seeking coping strategies (e.g., sharing stressful feelings with someone, asking for advice, etc.) between groups, qualitative analysis revealed that participants in the higher PTG group reported significantly more diverse support networks—not only close family but also extended social and professional resources—whereas those in the lower PTG group primarily relied on immediate family. This suggests that broader support engagement may facilitate or reflect greater PTG, as emotional disclosure and interpersonal sharing are known to promote key mechanisms of growth, including cognitive processing [6,51], dialectical reasoning [52], restructuring of core beliefs [53,54], and narrative reconstruction [55]. This finding underscores the importance of providing structured support and peer-sharing opportunities for parents during their child’s cancer recovery to facilitate adaptation [56].

Sociodemographic factors (e.g., marital status, parental age, number of children) and clinical variables (e.g., type of diagnosis, time since diagnosis, and recovery) were not significant predictors of parental PTG. However, career stability was associated with an increased likelihood of membership in the higher PTG class. Similarly, none of the psychosocial factors assessed following diagnosis—including perceived support, the child’s emotional and behavioral difficulties, parents’ initial stress responses, and family illness-related beliefs—were significantly associated with PTG latent class membership after treatment. However, parents’ initial stress reactions were associated with post-treatment PTSS and the neuroticism personality trait. These results are consistent with previous research indicating that psychological distress is associated with PTSS but does not predict PTG [57].

The small and heterogeneous nature of our sample, influenced by participant availability and self-selection into the second study phase, may have limited the detection of significant effects. Nevertheless, the lack of association between sociodemographic factors and PTG aligns with prior findings from both individual studies and systematic reviews [25]. For example, Barakat et al. [58] found no significant associations between sociodemographic variables and PTG among mothers, who are often the primary caregivers, consistent with our predominantly female sample (86%). Instead, psychosocial factors such as social support, active coping, optimism, and lower perceived illness impact have been more robustly linked to PTG and benefit finding [59], highlighting the central role of contextual and personal resources over static background characteristics.

### 4.3. Qualitative Indicators of Dynamic PTG Within a Mixed Methods Framework

Narrative analysis within the mixed methods framework revealed that parents in the higher PTG class exhibited elevated emotional awareness and self-reflection, as well as clearer indications of behavioral change. These participants frequently discussed emotional distress, side effects, and psychological processing while reflecting on the most traumatic moments during their child’s treatment, whereas parents with lower PTG scores often emphasized their shock and avoided emotional disclosure. Furthermore, narratives from the higher PTG group reveal a more pronounced awareness of bodily and emotional states, suggesting a form of somatic consciousness not previously emphasized in the existing PTG literature. Although parents in both groups reported significant physical stress following their child’s diagnosis, those in the higher PTG group demonstrated greater recognition and more vivid, open processing of these symptoms, which may support more effective adaptation to adversity [60,61].

Integrating a qualitative approach enabled parents to describe their transformative experiences without being primed to focus on growth, thereby revealing not only themes associated with PTG but also signs of internal strain, such as diminished self-trust due to emotional fatigue in contrast to perceived personal strength or social withdrawal occurring alongside deepened family bonds. While most themes reported by parents closely aligned with the five PTG domains proposed by Tedeschi and Calhoun [5], the domains of ‘New Possibilities’ and ‘Spiritual Growth’ were only sparsely represented in our sample’s qualitative data. ‘Appreciation of Life’ was the most represented domain in both groups’ narratives. Respondents with higher scores on the PTGI ‘Appreciation of Life’ subscale also consistently expressed a heightened awareness of everyday positives and a purposeful reordering of life priorities. This pattern may suggest that high appreciation scores are tied not merely to emotional relief but also to a fundamental reorientation of meaning and purpose following adversity.

A more nuanced reading of participants’ narratives reveals that increased appreciation is often accompanied by a deeper awareness of the seriousness and fragility of life, suggesting that gratitude and existential gravity can coexist within post-traumatic meaning-making [11]. As previously noted by Tedeschi et al., “understanding that symptoms of distress or disorder and PTG are not mutually exclusive requires dialectical thinking and appreciation of paradox. This is at the core of the PTG concept that loss can produce gain” [6] (p. 40). Our results support this view, as individuals in the higher PTG group reported not only indicators of growth but also significant emotional and physiological strain. This highlights the importance of moving beyond a binary framework of recovery and recognizing PTG as a dynamic process that often unfolds alongside ongoing struggle.

Beyond confirming Tedeschi and Calhoun’s model—originally developed from qualitative research, with PTGI items derived from participants’ own narratives—this study offers further nuance by identifying self-regulatory adaptations (e.g., reduced reactivity to everyday stressors resulting from shifts in value priorities and increased acceptance of life) as meaningful expressions of growth. Moreover, the emergence of prosocial motivation—a desire to support others through one’s own trauma—extends the ‘New Possibilities’ domain toward communal meaning-making. Although prosocial engagement following cancer-related trauma is well documented, its integration into the PTG framework highlights its role as a transformative coping strategy for individuals processing traumatic experiences [62].

### 4.4. Strengths and Limitations

This study contributes to the field of PTG research by reinforcing the notion that growth and psychological struggle can coexist and by highlighting that coping strategies, emotional processing, and personal meaning-making are more predictive of PTG than static sociodemographic characteristics or clinical variables. The mixed methods approach captured subtle dimensions of growth—particularly in bodily awareness, emotional restraint, and relational adaptation—that are not always measured in standardized PTG instruments. These insights underscore the importance of supporting cognitive–emotional integration and reflective processing during post-treatment survivorship care.

Future longitudinal research would benefit from larger, more representative samples to enhance generalizability; however, in the Latvian context, the sample used reflects the maximum feasible size given the limited number of newly diagnosed pediatric cancer cases annually. The current sample was predominantly composed of mothers (*n* = 50; 86%), which, while consistent with findings that mothers often serve as primary caregivers during a child’s hospitalization, limits the ability to draw conclusions about paternal experiences. Additionally, the exclusive reliance on self-report measures and a 51.7% attrition rate between T1 and T2 represent methodological limitations. Future studies should aim for more balanced gender representation among caregivers and consider incorporating multi-method approaches to strengthen the validity of their findings.

## 5. Conclusions

This mixed methods study identified culturally and contextually specific aspects of PTG among parents of CCSs that extend beyond what is typically captured by standardized instruments. The findings suggest that PTG is facilitated through processes such as engagement-based coping, self-reflection accompanied by shifts in value priorities, emotional expression, social sharing, and active involvement in psychological struggle rather than avoidance. This aligns with recent PTG models that frame growth as a dynamic integration of trauma, emotional regulation, and meaning-making rather than as a linear or purely positive transformation. As the first study to explore PTG in Latvian parents of CCSs, it addresses a significant gap in the literature and lays the groundwork for future culturally sensitive research.

Practice implications include the following: (1) strengthening engagement-based coping and emotional disclosure; (2) cultivating diverse support networks that extend beyond the immediate family to peers and professionals; and (3) acknowledging coexisting distress (e.g., fear of recurrence, somatic hypervigilance) while promoting value-guided regulation and acceptance. Mixed methods assessment can reveal nuanced, patient- and family-centered change processes that elude scale scores.

The findings underscore the importance of designing PTG interventions that strengthen emotional regulation, promote emotional disclosure as a means of externalizing psychological struggle, and foster self-reflection and constructive relational engagement (e.g., both accepting support and helping other parents with similar experiences). Crucially, such interventions should conceptualize psychological struggle not as a hindrance but as a potential catalyst for growth.

## Figures and Tables

**Table 1:** Descriptive statistics and coefficients of internal consistency for measures.

	*M*	*SD*	Range	Cronbach’s *α*
PTGI—Total	59.32	17.82	17–92	0.93
Relation to others	20.85	7.72	3–33	0.86
New possibilities	12.61	5.79	0–24	0.84
Personal strength	13.80	12.94	2–20	0.73
Spiritual change	5.23	3.34	0–10	0.82
Appreciation of life	11.50	12.86	0–15	0.84
IES-R—Total	1.81	0.57	0–3.49	0.92
Intrusion	1.81	0.66	0–3.61	0.84
Avoidance	1.82	0.64	0–3.71	0.84
Hyperarousal	1.86	0.55	0–3.33	0.65
RSQ				
Primary Control Engagement	23.55	4.08	15–36	0.79
Secondary Control Engagement	38.28	6.13	25–53	0.63
Disengagement	59.30	8.18	41–77	0.63
Involuntary Engagement	38.28	9.75	18–60	0.81
Involuntary Disengagement	25.12	6.97	12–39	0.81

PTGI—Post-traumatic Growth Inventory; IES-R—Impact of Event Scale-Revised; RSQ—Responses to Stress Questionnaire.

**Table 2:** Spearman’s correlation between outcome measures at T2 and variables measured at T1.

	Measures at T1						
	N	E	O	A	C	Stress Reactions	Family Beliefs
*Measures at T2* IES-R—Total	0.36 **	−0.19	−0.23	−0.19	−0.01	0.41 **	0.22
Intrusion	0.37 **	−0.17	−0.27 *	−0.08	−0.05	0.45 ***	0.20
Avoidance	0.23	−0.22	−0.18	−0.21	0.02	0.33 *	0.25
Hyperarousal	0.34 *	−0.05	−0.13	−0.17	−0.01	0.38 **	0.16
PTGI—Total	−0.09	0.07	0.10	0.33 *	0.18	−0.16	−0.39 **
Relation to others	0.11	0.02	0.16	0.17	−0.07	−0.05	−0.32 *
New possibilities	−0.08	0.07	0.14	0.24	0.12	−0.12	−0.30 *
Personal strength	−0.45 **	0.22	0.09	0.43 **	0.25	−0.30 *	−0.43 **
Spiritual change	0.07	−0.13	−0.15	0.12	0.15	−0.02	−0.19
Appreciation of life	0.03	−0.01	−0.01	−0.05	−0.01	0.01	−0.15

IES-R—impact of event scale-revised; PTGI—Post-traumatic Growth Inventory; N—neuroticism, E—extraversion, O—openness, A—agreeableness, C—conscientiousness. * *p* < 0.05. ** *p* < 0.01. *** *p* < 0.001.

**Table 3:** Bayesian logistic regression estimates of coping strategies predicting class membership.

Coping Strategy (RSQ)	Estimate	Est. Error	Lower (95% CI)	Upper (95% CI)
Primary Engagement	−0.16	0.08	−0.33	−0.01
Secondary Engagement	−0.07	0.08	−0.22	0.08
Disengagement	0.03	0.05	−0.07	0.13
Involuntary Engagement	0.02	0.03	−0.05	0.09
Involuntary Disengagement	0.04	0.05	−0.05	0.13

RSQ—Responses to Stress Questionnaire.

**Table 4:** Bayesian logistic regression estimates of psychosocial factors predicting latent class membership.

Subscale of PAT	Estimate	Est. Error	Lower (95% CI)	Upper (95% CI)
Social Support	0.05	0.06	−0.06	0.17
Child Problems	−0.07	0.08	−0.23	0.09
Family Problems	0.18	0.30	−0.41	0.78
Stress Reactions	0.03	0.04	−0.05	0.12
Family Beliefs	0.01	0.10	−0.17	0.21

PAT—Psychosocial Assessment Tool.

**Table 5:** Post-traumatic growth domains and thematic frequencies in latent classes.

PTG Domain	Themes Reflecting Changes	G1 *Prop.*	G2 *Prop.*	Examples
**Relating to Others**	Improved Family Bonds/ Change in Priorities	*0.16* *(6)*	*0.10* *(2)*	*I value the time spent with my family. (G1, 50A)* *I try to work less and spend more time with my family. (G2, 51A)*
	Increased Empathy and Connection	*0.05* *(2)*	*-*	*I became more inwardly reserved yet simultaneously more empathetic and emotionally calm. (G1, 32B)*
	Social Withdrawal/ Trust Shift (Negative)	*0.05* *(2)*	*0.05* *(1)*	*Strangers feel like a threat, acquaintances feel safe. It wasn’t like that before. (G1, 15B)* *I live in my own world. (G2, 24B)*
	Relational Strain (Negative)	*0.08* *(3)*	*-*	*It’s hard for me to listen to other people’s problems, which I wouldn’t call problems anymore. This change might damage relationships. (G1, 63B)*
**New Possibilities**	Behavioral Adaptation and Self-Care Awareness	*0.05* *(2)*	*-*	*I pay more attention to children’s health, try to spend more time with them. (G1, 27B)*
	Desire to Help Others Through Experience (emerging theme)	*-*	*0.10* *(2)*	*I want my experience to help someone else,* e.g., *through conversations with those currently undergoing treatment. (G2, 21A)*
	Changes in Daily Routine (Negative)	*0.03* *(1)*	*-*	*I sense physical inactivity, slight irritation about my weight, and drowsiness. My child and work are priorities now. I miss time alone. (G1, 48B)*
**Personal Strength**	Self-Efficacy and Inner Strength	*0.08* *(3)*	*-*	*Illness showed me how strong I really am! (G1, 40B)*
	Increased Expression/Assertiveness	*0.05* *(2)*	*-*	*I express my opinion more. (G1, 14A)*
	Emotional Maturity and Regulation	*0.19* *(7)*	*0.05* *(1)*	*I’ve become calmer about the big things in life because I’m no longer as afraid. (G1, 31B)* *I think I’m the same person overall, just more hardened. (G2, 16B)*
	Reduced Reactivity to Trivial Matters	*0.24* *(7)*	*0.14* *(3)*	*I don’t worry about little things. Less interest in the insignificant. (G1, 28B)* *I don’t stress about unimportant things. (G2, 17B)*
	Increased Anxiety/Stress (Negative)	*0.05* *(2)*	*0.19* *(4)*	*I worry more, I listen to my inner voice and feelings. (G1, 17A)* *Lower stress tolerance; even unrelated stress triggers bodily reactions faster. (G2, 21A)*
	Health Hypervigilance (Negative)	*0.03* *(1)*	*0.10* *(2)*	*Cautious, more responsible. (G1, 50A)* *I react paranoidly to every complaint from my child. (G2, 9A)*
	Emotional Changes (Negative)	*0.11* *(4)*	*0.10* *(2)*	*I have become angrier, quicker to snap. (G1, 29A)* *Less joyful attitude toward life. (G2, 33B)*
	Ongoing Psychological Burden (Negative)	*0.03* *(1)*	*0.14* *(3)*	*Extreme fatigue has built up. (G1, 46B)* *I began experiencing insomnia—which I hadn’t had before—and started using antidepressants. (G2, 18A)*
**Spiritual Change**	Spiritual Growth/ Trust in God	*0.05* *(2)*	*0.05* *(1)*	*I started to believe in God and feel thankful for the good people in life. (G1, 40B)* *I’m confident and fully convinced of God’s love and the medical quality in Latvia. (G2, 4A)*
**Appreciation of** **Life**	Gratitude and Appreciation of Life	*0.24* *(9)*	*0.10* *(2)*	*I see everything around me as a gift. (G1, 40B)* *I’m more appreciative of what I have. (G2, 7A)*
	Perspective Shift/ Change in Values	*0.22* *(8)*	*0.24* *(5)*	*I’ve come to understand what truly matters in life and don’t focus on external problems. (G1, 55B)* *Reviewing my values (want to reduce the meaningless elements of life). (G2, 21A)*
	Mindfulness/ Living in the Moment	*0.14* *(5)*	*0.05* *(1)*	*I accept each moment and day as a gift, try not to plan because I don’t know what tomorrow brings. (G1, 12A)* *I try to work less and enjoy the moment with family. (G2, 51A)*

G1—higher PTG group; G2—lower PTG group; prop.—proportional mean.

**Table 6:** Joint display of quantitative, qualitative, and mixed methods meta-inferences of PTG domains.

PTG Domain	Boxplots of Group-Based Variations in PTGI Subscales	Qualitative Subcategories with Examples	Mixed Methods Comparison
**Relating to Others**	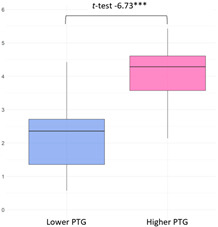	Improved Family Bonds/Change in Priorities (8 narratives) *My life priorities have changed—I value family more than my career. (G2, 30A)* *I appreciate the seemingly obvious things much more, the genuine people around me; I love and protect those who are mine even more. (G1, 32B)* *Complete indifference to what others think or might think of me (this is both positive and negative). I value helpful people much more than I did before. (G1, 28A)* Increased Empathy and Connection (2) *I have become more sensitive. […] [and] rely more on those around me. (G1, 29B)* *I became more inwardly reserved yet simultaneously more empathetic and emotionally calm. (G1, 32B)*	In the lower PTG group (M = 2.25, SD = 1.16), growth within the ‘Relating to Others’ domain showed greater variability, suggesting divergent relational experiences following adversity. Narratives from participants in both groups pointed to a reorientation of relational priorities, characterized by a renewed focus on close family ties and a heightened appreciation for genuinely supportive individuals. Quantitatively, this nuance is reflected in the pattern of item responses: participants reported higher agreement with PTGI item 15 (“*I have more compassion for others*”), yet lower scores on item 9 (“*I am more willing to express my emotions*”). This discrepancy suggests that while empathic concern may have increased, emotional openness remained constrained.
**New Possibilities**	** 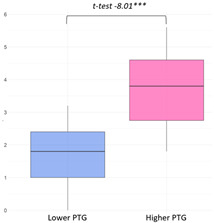 **	Behavioral Adaptation and Self-Care Awareness (2) *I pay more attention to children’s health, try to spend more time with them. (G1, 27B)* Desire to Help Others Through Experience (2) *I want my experience to help someone else,* e.g., *through conversations with those currently undergoing treatment. (G2, 21A)* *I would like to share our family’s experience with other parents as a source of support and encouragement. (G2, 12A-2)*	The marked variability in ‘New Possibilities’ scores suggests ambivalence toward transformative growth. Narrative representation was limited. The prosocial theme—‘Desire to Help Others Through Experience’—unexpectedly emerged only from the lower PTG group. The lower endorsement of items related to new interests and opportunities contrasts with higher agreement on pragmatic change (item 17: “*I am more likely to try to change things that need changing*”), indicating a preference for practical adaptation over personal reinvention.
**Personal Strength**	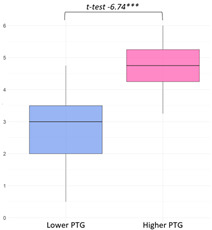	Self-Efficacy and Inner Strength (3) *I have found the strength within myself to ask for help when I need it. (G1, 20B)* Increased Expression/Assertiveness (2) *I’ve become much braver, more assertive, and more demanding (G1, 32B)* Emotional Maturity and Regulation (8) *I am more responsible, more mature, more logical now. (G1, 9B)* Reduced Reactivity to Trivial Matters (10) *I don’t stress about my child’s tantrums and tricks; I let them act out, and then we talk everything through afterward. (G1, 19A)*	Parents with higher ‘Personal Strength’ scores on the PTGI often described cognitive–emotional growth in their narratives, particularly in the ‘Personal Strength’ and ‘Relating to Others’ domains. The theme ‘Reduced Reactivity to Trivial Matters’ also aligns with ‘Perspective Shift/Change in Values’ from the ‘Appreciation of Life’ domain, suggesting interconnected changes across domains. In the ‘Personal Strength’ domain, reported growth was frequently accompanied by ongoing anxiety, increased health vigilance, and lasting psychological burden. These patterns appeared more prominently among individuals in the lower PTG group.
**Spiritual Growth**	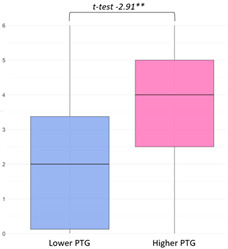	Spiritual Growth/Trust in God (3) *Modesty, work, and career are not the most important things in life. (G1, 55A)* *I started to believe in God and feel thankful for the good people in life. (G1, 40B)* *I’m confident and fully convinced of God’s love and the quality of Latvian medical care. (G2, 4A)*	The ‘Spiritual Change’ domain exhibited the greatest score dispersion among all PTGI subscales. Despite this variability, the difference between the higher and lower PTG groups remained significant, with the lower PTG group showing greater variability, suggesting potential spiritual ambivalence or strain. Narrative representation was limited. Quantitatively, parents more frequently endorsed an increased “*understanding of spiritual matters*” (item 5), while the “*strengthening of religious faith*” (item 18) was less commonly reported, indicating a broader, more individualized interpretation of spiritual rather than religious growth.
**Appreciation of Life**	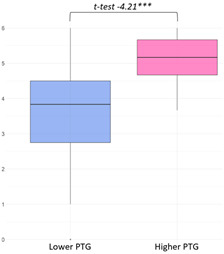	Gratitude and Appreciation of Life (11) *I see everything around me as a gift. (G1, 40B)* *I’m much better at appreciating life’s small positive moments! (G1, 19A)* Perspective Shift/Change in Values (13) *A completely different outlook on life. Different thoughts about people. (G1, 40A)* *I’ve realized what truly matters in life. (G1, 55B)* Mindfulness/Living in the moment (6) *The ability to find joy in everyday things and to live in the present moment. (G1, 12B)* *I live for today. (G1, 55B)*	‘Appreciation of Life’ was the most frequently represented domain across narratives in both groups. High scorers consistently described a heightened awareness of everyday positives and a shift in life priorities, reflecting not just emotional relief but a deeper reorientation of meaning and purpose. However, such responses warrant cautious interpretation, as social desirability and illusory growth may influence self-reports. Importantly, narratives also revealed that gratitude often coexisted with a sharpened sense of life’s fragility, underscoring the complex interplay between appreciation and existential awareness in PTG.

** *p* < 0.01. *** *p* < 0.001.

## Data Availability

The raw data supporting the conclusions of this article will be made available by the authors, without undue reservation.
